# Hormonal Management for the Induction of Luteolysis and Ovulation in Andalusian Jennies: Effect on Reproductive Performance, Embryo Quality and Recovery Rate

**DOI:** 10.3390/ani12020143

**Published:** 2022-01-08

**Authors:** Marina Bottrel, Isabel Ortiz, Manuel Hidalgo, María Díaz-Jiménez, Blasa Pereira, César Consuegra, Mohamed Samy Yousef, Jesús Dorado

**Affiliations:** 1 Veterinary Reproduction Group, Department of Animal Medicine and Surgery, Faculty of Veterinary Medicine, University of Cordoba, 14014 Cordoba, Spain; ma.bottrel@gmail.com (M.B.); iortiz@uco.es (I.O.); mhidalgo@uco.es (M.H.); mariadijim@gmail.com (M.D.-J.); bpaguilar93@gmail.com (B.P.); cconsuegra93@hotmail.com (C.C.); 2 Department of Theriogenology, Faculty of Veterinary Medicine, Assiut University, Assiut 71515, Egypt; elmaamly20102002@yahoo.com

**Keywords:** ovulation, luteolysis, embryo recovery rate, embryo quality, embryo donor, jennies, donkey embryo

## Abstract

**Simple Summary:**

Embryo transfer is of utmost importance in endangered species such as donkeys. To maximize the number of embryos obtained and, thus, the number of foals born, the manipulation of cycles (shortening of interval between cycles and timely ovulation induction) is crucial. Therefore, the aim of this study was to compare two prostaglandins (luprostiol, LUP, and dinoprost, DIN) and two ovulation-inducing agents (human Chorionic Gonadotropin, hCG, and deslorelin, DES) in 26 fertile embryo donor jennies. In Experiment 1, jennies were randomly treated with either LUP or DIN after recovering the embryos. In Experiment 2, the jennies were treated with either hCG or DES to synchronize ovulation and breeding. Both prostaglandins shortened the cycles similarly and did not affect the embryos recovered. Although the ovulation inductors reported similar results, DES induced ovulation more rapidly. Interestingly, the embryo quality was lower when the uterus showed high edema at the time of insemination.

**Abstract:**

Two prostanglandins (luprostiol, LUP, and dinoprost, DIN) and two ovulation-inducing agents (human Chorionic Gonadotropin, hCG, and deslorelin, DES) were evaluated for luteolysis and estrus induction, and for ovulation induction, respectively, in embryo donor jennies. Twenty-six fertile Andalusian jennies were used. In Experiment 1, jennies (*n* = 112 cycles) were randomly treated with either LUP or DIN after embryo flushing. In Experiment 2, donors (*n* = 84 cycles) were randomly treated with either hCG or DES to induce ovulation. No differences were found between prostaglandins for all variables studied (prostaglandin–ovulation interval (POI), interovulatory interval (IOI), embryo recovery rate (ERR), positive flushing rate (PFR) and embryo grade (EG)). The ovulation rate was similar for hCG and DES (60.9% vs. 78.7%). However, the interval to ovulation (ITO) was affected (62.61 ± 7.20 vs. 48.79 ± 2.69 h). None of the other variables studied (ERR, PFR and EG) were affected (*p* > 0.05), except for embryo quality (*p* = 0.009). In short, both prostaglandins evaluated are adequate to induce luteolysis and estrus. Both ovulation-inducing agents hastened ovulation, but DES seems to be more effective than hCG. Follicular diameter affected the interval from treatment to ovulation, and high uterine edema was related to low embryo quality.

## 1. Introduction

Not only is the application of assisted reproduction techniques (ARTs), such as embryo transfer (ET), artificial insemination (AI) and hormonal treatments, in donkeys possible [1,2,3,4], but it can also improve the reproductive efficiency and maximize offspring production [5]. These goals are particularly important for the preservation of endangered breeds such as the Andalusian donkey (*Equus asinus africanus*), which is listed as a breed in danger of extinction [6] due to its small population size [7]. In this context, manipulation of the jennies’ estrous cycle is a crucial tool to increase the breeding efficiency in donkeys. Unfortunately, the number of studies on this species is limited [8,9,10,11,12] in comparison with the horse. In addition, the response to pharmacological therapy for the induction of luteolysis and ovulation varies among individuals of this species [13].

Luteolytic drugs, such as prostaglandin F2alpha (PGF2α) and its synthetic analogues (PGF analogues), are commonly used for shortening the estrous cycle in mares, by decreasing diestrus and, consequently, the interovulatory interval (IOI), without affecting the fertility of the treated animals [14]. Although the length of estrus is similar between jennies and mares, the duration of diestrus is longer in jennies [11,15]. Therefore, the use of luteolytic hormones becomes an essential tool to increase the number of collected embryos from a donor. Several luteolytic agents have been successfully used in jennies [16,17,18]; of these, the most frequently used are dinoprost tromethamine (as PGF2α) and cloprostenol and luprostiol (as PGF analogues). The main advantages of synthetic analogues over natural prostaglandins are their longer half-life, effectiveness at lower dosages and fewer undesirable side effects [19]. The administration of a prostaglandin (PG) to mares with a mature corpus luteum (CL) reduces the progesterone concentration within 36 h [20] and induces estrus 2–5 days after administration [14]. However, the interval from PG treatment to ovulation (PG–ovulation interval, POI) is greatly variable (2–16 days) [21,22], regardless of the day of the estrous cycle in which the treatment is administered [20]. Hence, it is known that the main factors that influence the POI after PG administration are dosage, follicular diameter at the time of treatment and follicular status (growing vs. atresia) [14,23,24]. Despite the numerous studies carried out on mares, a very limited number of studies were found in the literature on the effects of the administration of PG in jennies, and these few studies showed that the POI did not vary when PGF2α was administered at different days after ovulation (POI: 9.2–10.2 days) [9,18,25].

The induction of ovulation in a short predictable time (within 48 h after treatment) becomes useful not only to reduce the duration of estrus and the number of inseminations or matings per cycle but also to have a closer synchronization between insemination and ovulation [26,27]. In addition, ovulation management is an important tool to synchronize the donor’s and recipient’s cycles in ET programs [4,28,29]. There are several hormones that can be used to induce ovulation in equine species [30,31], such as human Chorionic Gonadotropin (hCG), Equine Pituitary Extract (EPE) and Gonadotropin-Releasing Hormone (GnRH). Although the efficacy of both EPE and GnRH in hastening ovulation has been widely proven in mares [26,28,30,32,33,34], hCG is still the most popular inductor, not only in mares [27,30,31], but also in jennies [35,36], due to its high efficacy, easier commercial availability and lower cost. However, various studies have shown that repeated administration of this hormone can stimulate the synthesis of anti-hCG antibodies, leading to a loss of its effectiveness [37,38]. Hence, it is usually not recommended to administer hCG more than twice within the same breeding season [39], which is not practical in ET programs. Luckily, GnRH agonists can be administered repeatedly [40], becoming the preferred alternative to hCG for timed ovulation induction. In mares, the GnRH agonists buserelin acetate and deslorelin acetate were proven to be able to induce ovulation 24–48 h after administration, similarly to hCG [41,42,43]. Moreover, a more reliable induction of ovulation has been suggested when deslorelin acetate is administered to mares that have large thick-walled follicles in comparison to hCG [44,45,46,47]. However, only a few studies have been performed on the use of a single administration of either hCG [35], lecirelin acetate [35], buserelin acetate [3] or deslorelin acetate [48] as ovulation-inducing agents in jennies. There are several factors related to success when using ovulation-inducing agents. The follicular diameter of the largest follicle at the moment of treatment with hCG and GnRH analogues has been previously demonstrated in mares [27,32] and donkeys [49]. It is also known that the uterine edema pattern depends on the prevailing circulating levels of ovarian steroids [50], increasing edema scores as estrogens rise. More recently, it has been suggested that the failure of LH to induce ovulation in mares might be due to the absence of estradiol-positive feedback [51]. In mares, both the preovulatory follicular diameter and uterine edema pattern at the time of treatment may be used to predict the response to ovulation-inducing treatment [52]. However, to the best of our knowledge, there are no studies on this issue performed on donkeys.

The main objectives of the present study were to: (i) evaluate the efficacy of two luteolytic agents, luprostiol (as a PGF analogue) and dinoprost tromethamine (as PGF2α), for luteolysis and estrus induction in donor jennies, and (ii) compare the efficacy of hCG and deslorelin acetate in inducing ovulation of embryo donor jennies. Further objectives of this study were to: (iii) examine the effects of either the prostanglandins or ovulation-inducing agents on the embryo recovery rate (ERR) and embryo morphological quality (as embryo grade score (EG)), and (iv) determine whether follicular diameter and uterine edema score at the time of treatment affect reproductive performance, ERR and EG in the treated donor jennies.

## 2. Materials and Methods

### 2.1. Animals, Ultrasound Examination and Breeding

This study was carried out at the Malpica Rural Center (Palma del Río, Cordoba, Spain). A total of twenty-six fertile Andalusian jennies (3–13 years old) were used as embryo donors between February 2015 and December 2017. All the jennies were non-lactating, clinically healthy and in good body condition, kept in outdoor parks and provided with water ad libitum, teff hay and oats.

Follicular development, degree of uterine edema and ovulation were monitored by trans-rectal ultrasonography (Aloka SSD 500, 7.5 MHz linear probe, ALOKA Co., Ltd., Tokyo, Japan). The diameter of the largest follicle(s) was measured with electronic calipers [53], and uterine edema was scored on a scale from 0 to 4, where the score of 0 corresponded to no ultrasonographic presence of endometrial folds, and a score of 4 was assigned to jennies with maximal endometrial folding [52]. Examinations were carried out twice a week, changing to every 24 h after the largest follicle reached 25 mm in diameter, until ovulation was confirmed. Records of 133 estrous cycles were analyzed retrospectively.

When a follicle of 35–40 mm was detected in the presence of uterine edema, jennies were randomly assigned to receive an ovulation-inducing drug (hCG or GnRH analogue, according to the experimental design). Donor jennies were mated naturally with a fertile jack every other day until ovulation was detected (Day 0).

### 2.2. Embryo Recovery

Embryos were collected between days 6 and 9 post-ovulation by transcervical flushing of the uterus using up to 3 × 1 L of Lactated Ringer’s solution, as described by Camillo et al. [4] for donkeys. Recovered embryos were washed ten times in Syngro^®^ Holding, as previously described [1], then evaluated for morphological quality and graded on a modified four-point scale (Grade 1: excellent; Grade 2: good; Grade 3: fair; Grade 4: poor, degenerate or dead) [54]. After each uterine flushing, an intramuscular (i.m.) dose of prostaglandin was applied (PGF analogue or PGF2, according to the experimental design) to induce the lysis of the CL. A total of 133 embryo flushings were included in the present study.

### 2.3. Experimental Design

#### 2.3.1. Experiment 1: Comparison of Luprostiol and Dinoprost Tromethamine for Luteolysis and Estrus Induction in Jennies

In total, 112 estrous cycles of 19 jennies were randomly assigned to two groups (no particular order of administration of products was followed): (i) LUP (*n* = 79 cycles): treated with a single i.m. injection of 5.25 mg of luprostiol (Prosolvin^®^, Virbac, Barcelona, Spain), and (ii) DIN (*n* = 33 cycles): treated with a single i.m. injection of 3.5 mg of dinoprost tromethamine (Lutalyse^®^, Pfizer Animal Health, São Paulo, Brazil). The prostaglandin–ovulation interval (POI; interval in days from drug administration to ovulation) and the interovulatory interval (IOI; period in days between two ovulations) were recorded. For data analysis purposes, POI was classified into 3 categories: (i) <9 days, (ii) 9–11 days and (iii) >11 days. The embryo recovery rate (ERR; embryos recovered per cycle), embryo grade (EG) and positive flushing rate (PFR; flushing where at least one embryo was recovered) were also investigated.

#### 2.3.2. Experiment 2: Comparison of hCG and Deslorelin Acetate for Ovulation Induction in Jennies

Jennies in estrus (19 jennies and 84 cycles) were randomly assigned to two groups (no particular order of administration of products was followed): (i) hCG (*n* = 23 cycles): treated with a single i.m. injection of 1500 IU of hCG (Veterin Corion^®^, Divasa Farmavic, Barcelona, Spain), and (ii) DES (*n* = 61 cycles): treated with a single i.m. injection of 0.75 mg deslorelin acetate (Sincrorrelin^®^, Ourofino Saúde Animal, Cravinhos, Brazil). The interval to ovulation (ITO, interval in hours from drug administration to subsequent ovulation) was recorded. A positive response to the ovulation-inducing treatments was defined as follows: ovulation occurred 24–48 h after the administration of the treatment [3]. In both treatment groups (hCG and DES), follicles were grouped into 3 categories based on the diameter (≤35 mm, 36–39 mm and ≥40 mm), while uterine edema was assigned a score of 0–4. The ovulation rate (OR; ovulations per cycle), ERR, EG and PFR were also recorded.

### 2.4. Statistical Analysis

Normality was tested using the Kolmogorov–Smirnov test. Differences between groups in percentage of jennies ovulating within 48 h, percentage of double ovulations, ovulation rate, embryo collection rate and positive uterine flushing rates were evaluated by Fisher’s exact test. Continuous variables (age, follicular diameter at time of injection of ovulation-inducing treatment, prostanglandin–ovulation interval, interovulatory interval and interval to ovulation) were analyzed using Levene’s test for homogeneity of variance and a Kruskal–Wallis test. A Mann–Whitney U test was used to evaluate the uterine edema score at the time of treatment and embryo morphological grade. The correlation between preovulatory follicle diameter at the time of treatment and the uterine edema score at the time of treatment and interval to ovulation was assessed by Spearman’s correlation analysis. The statistical analysis was carried out using the SPSS v15.0 statistical package (IBM Spain, Madrid, Spain). The differences were considered statistically significant when *p* < 0.05. Data are shown as percentage (relative frequency) or mean ± standard error of the mean (SEM), as appropriate.

## 3. Results

### 3.1. Experiment 1: Comparison of Luprostiol and Dinoprost Tromethamine for Luteolysis and Estrus Induction in Jennies

As shown in Table 1, LUP was administered to 16 jennies during 79 cycles, and DIN was administered to 14 jennies during 33 cycles. The mean age of the jennies did not differ (*p* = 0.862) between treatment groups (6.87 ± 0.37 in LUP vs. 6.76 ± 0.52 in DIN). There was no difference between treatment groups for POI (9.73 ± 0.33 vs. 10.21 ± 0.57; LUP vs. DIN; *p* = 0.448) and IOI (16.86 ± 0.34 vs. 17.73 ± 0.55; LUP vs. DIN; *p* = 0.175).

Table 2 shows that there were no differences (*p* > 0.05) among seasons in POI or IOI in jennies treated with LUP or DIN. However, in winter, mean POI values were significantly higher (*p* = 0.021) in DIN-treated (11.40 ± 0.75 days) than in LUP-treated jennies (8.88 ± 0.58 days).

As shown in Table 3, the response to LUP was significantly higher (*p* = 0.0166) during the interval between 9 and 11 days (40.5%) than after 11 days (22.8%). In contrast, no differences (*p* > 0.05) were observed in the distribution of estrous cycles in DIN-treated jennies. For each time period (<9 days, 9–11 days and >11 days), no effect of treatment was observed (*p* > 0.05; Table 3).

As shown in Table 4, 112 uterine flushings were carried out during this experiment, of which 72 were positive (PFR: 64.3%), and 78 embryos were recovered out of 112 estrous cycles (ERR: 69.6%). PFR, ERR and EG were not significantly (*p* > 0.05) different between PG treatment groups. However, both PFR (72.7% vs. 60.8%; *p* = 0.2282) and ERR (81.8% vs. 64.6%; *p* = 0.0701) showed numerically higher values in DIN-treated than in LUP-treated jennies (Table 4).

### 3.2. Experiment 2: Comparison of hCG and Deslorelin Acetate for Ovulation Induction in Jennies

As shown in Table 5, hCG was administered to 13 jennies during 23 cycles, and DES was administered to 12 jennies during 61 cycles. There was no significant difference (*p* > 0.05) in the age of the jennies between the two treatment groups (6.83 ± 0.47 years vs. 6.56 ± 0.45 years; hCG vs. DES). Similarly, the average follicular diameter at the time of treatment did not differ between hCG-treated and DES-treated jennies (37.85 ± 1.27 mm vs. 36.34 ± 0.71 mm; hCG vs. DES; *p* = 0.732). However, jennies treated with hCG had a higher (*p* = 0.022) uterine edema score at the time of treatment as compared with DES-treated jennies (2.56 ± 0.17 vs. 2.00 ± 0.12; Table 5). In addition, ITO was significantly higher in hCG-treated than in DES-treated jennies (62.61 ± 7.20 h vs. 48.79 ± 2.69 h; *p* = 0.029). Nevertheless, there was no difference (*p* > 0.05) in the percentage of cycles in which ovulation occurred within 48 h after administration of hCG as compared with DES (60.9% vs. 78.7%; hCG vs. DES). Likewise, no differences (*p* > 0.05) in the percentage of double ovulations were observed between hCG-treated and DES-treated jennies (39.1% vs. 24.6%; hCG vs. DES; Table 5).

As shown in Table 6, the DES-induced ovulation rate was significantly higher 24–48 h after DES administration (54.1%) than in the initial 24 h (24.6%; *p* = 0.0009) or 48 h after (21.3%; *p* = 0.0002). A similar numerical tendency was observed in hCG-induced jennies (17.4%, 43.5% and 39.1%, respectively), although no significant differences were found (*p* = 0.0545; Table 6). There were no significant differences (*p* > 0.05) for ovulation rates within each time period (0–24 h, 24–48 h and >48 h) between the two treatments (hCG vs. DES). Failure of ovulation within 48 h after administration of an ovulation-inducing agent occurred in 22 of 84 cycles (26.2%), which included 9 cycles treated with hCG (39.1%) and 13 with DES (21.3%). Of the nine hCG-treated jennies that failed to ovulate within 48 h, five (55.6%) jennies ovulated by 72 h, one (11.1%) by 96 h and three (33.3%) > 120 h after hCG administration. Of the 13 DES-treated jennies that failed to ovulate within 48 h, 11 (84.6%) jennies ovulated by 72 h, 1 (7.7%) by 96 h and 1 (7.7%) > 120 h after DES administration. Nonresponder jennies (ITO > 48 h) were treated, on average, at a numerically smaller follicular diameter than those that responded to hCG or DES (34.75 ± 1.22 mm vs. 37.47 ± 0.71 mm; *p* = 0.051). Interestingly, the average preovulatory follicle diameter in the cycles in which no ovulation-inducing agent was used (39.89 ± 0.91 mm) was significantly higher (*p* = 0.012) than in the DES-treated cycles (36.34 ± 0.71 mm). The preovulatory follicle size in hCG-treated cycles was not significantly different (37.85 ± 1.27 mm; *p* > 0.05) from either the non-treated or DES-treated cycles. In contrast, the uterine edema score at the time of treatment was significantly higher (*p* = 0.040) in nonresponder jennies than in hCG-treated or DES-treated jennies (2.48 ± 0.20 vs. 2.00 ± 0.12).

Regarding seasonality, there was no effect (*p* > 0.05) of season on ITO in jennies treated with either hCG or DES (Table 7). However, in DES-treated jennies, the ovulation rate was significantly affected by season (*p* < 0.05), being higher in summer and autumn than in winter (88.9% vs. 33.3%). On the contrary, no effect was noticed in hCG-treated jennies (*p* > 0.05; Table 7).

A total of 84 flushings were carried out during the experimental period (Table 8), of which 55 were positive (PFR: 65.5%), and 58 embryos were recovered out of 84 estrous cycles (ERR: 69.0%). There were no significant (*p* > 0.05) differences between the hCG and DES groups for PFR, ERR and EG. Nevertheless, numerically higher (*p* > 0.05) percentages of PFR and ERR were obtained in DES-treated than in hCG-treated jennies (52.2% vs. 70.5% and 72.1% vs. 60.9%, respectively).

Table 9 shows that ITO increased (*p* = 0.005) as the follicular diameter decreased, being higher (64.30 ± 5.54 h) when the ovulation-inducing agent was administered in the presence of follicles ≤ 35 mm. Moreover, a negative relationship was detected between follicular diameter at the time of treatment and ITO (r = −0.357; *p* < 0.001). However, follicular diameter at the time of treatment did not influence (*p* > 0.05) PFR, ERR or EG (Table 9).

As shown in Table 10, no influence of the uterine edema score at the time of treatment on ITO was detected (*p* > 0.05). However, a positive relationship between both variables was observed (r = 0.262; *p* < 0.021). No significant differences (*p* > 0.05) between degrees of uterine edema were observed for PFR and ERR. However, the quality of the embryo significantly (*p* = 0.009) decreased for a uterine edema grade ≥ 3 (1.71 ± 0.16) in comparison to lower grades (0, 1 and 2; Table 10).

## 4. Discussion

To the best of our knowledge, this is the first study in which the effects of the prostaglandin agents luprostiol (LUP) and dinoprost tromethamine (DIN) are compared in jennies. According to a previous study in mares [55], these luteolytic drugs had a similar luteolytic effect, since the interval from injection to detection of ovulation (POI) was not significantly different between PG treatments. In addition, similar mean values of IOI (interovulatory interval) were found for LUP-treated and DIN-treated jennies.

Luprostiol has previously been used in Andalusian jennies [18]. In that study, POI (10.22 ± 0.92 days) and mainly IOI (23.07 ± 0.50 days) were slightly higher than our results (POI: 9.73 ± 0.33 days; IOI: 16.86 ± 0.34 days). As reported previously in mares [20,22], a high variation in the POI of jennies was observed (LUP: from a minimum of 6 days to a maximum of 20 days; DIN: from a minimum of 4 days to a maximum of 17 days), which could be caused by a dose effect [24,55] rather than by the day of diestrus in which the PGF analogue is administered [23]. In contrast to a previous study on diestrus that used 7.5 mg lupostriol [18], a lupostriol dosage of 5.25 mg on days 6–9 of diestrus was used in our study. It is also known that POI mainly depends on the dominant follicle size at the time of treatment and on the subsequent follicular development and estrogen production [23,24], which could be different between studies, complicating the comparison of findings [9]. Regarding DIN, our results are in consonance with those reported by Goretti et al. [5] in donor mares treated with dinoprost tromethamine, in which POI and IOI were 10.0 ± 0.9 days and 17.5 ± 1.1 days, respectively.

On the other hand, both PG treatments (LUP and DIN) were able to reduce the diestrus length as IOI was shortened, on average, to 17.12 ± 0.29 days (LUP: 16.86 ± 0.34 days; DIN: 17.73 ± 0.55 days), thereby improving the reproductive performance of donor jennies in ET programs. However, when the distribution of estrous cycles was examined, it was noteworthy that the induction of estrus with LUP resulted in a better synchronization of estrus and ovulation than treatment with DIN. Thus, the proportion of LUP-treated jennies with a POI of greater than 11 days was significantly lower than that of the group with a POI of 9 to 11 days (22.8% vs. 40.5%), but this finding was not observed in DIN-treated jennies. Differences in the response between PGF2α and the PGF analogue could be due to a stimulatory effect of the PGF analogue on the hypothalamus and pituitary, as has already been reported in luprostiol-treated mares during the spring transitional period [56]. This study also reported that luprostiol causes a transient increase in FSH and LH concentrations in jugular blood, thereby resulting in direct induction of ovulation.

Similar to previous studies performed on luprostiol-treated jennies [18] and on cloprostenol-treated mares [57], no effect of season on both POI and IOI was observed. However, it is noteworthy that during winter, POI was significantly shorter in LUP-treated than in DIN-treated jennies. Follicular diameter at the time of treatment was higher in LUP-treated than in DIN-treated jennies during winter (38.78 ± 2.06 mm vs. 35.00 ± 0.00 mm), which could explain our results as the largest follicle at the time of treatment might have a higher daily growth rate [23,24].

The overall ERR obtained in this study (69.3%) was similar to that reported in the literature for donkeys (40.7–80.6%) [4,58,59,60,61]. Our results do not show any significant differences between PG treatments for PFR, ERR and EG, which is consistent with previous reports on mares [23]. However, in our opinion, statistical differences could not be determined because of the small subgroup sizes. Thus, the overall ERR tended to be higher (*p* = 0.0701) in DIN-treated than in LUP-treated jennies (81.8% vs. 64.6%), without affecting the embryo morphological quality (i.e., EG).

The ovulation rate within 48 h is generally considered as an accurate criterion for evaluating the ability of a treatment to induce ovulation [62]. No differences between ovulation-inducing treatments (hCG and DES) were observed for this parameter, which is consistent with previous studies in mares [63,64,65,66] and jennies [35]. However, as previously reported [52], it seems probable that DES has a better ability to induce ovulation of smaller follicles than hCG and thereby affect their efficacy (DES: 78.7% vs. hCG: 60.9%; *p* = 0.086). DES-treated jennies ovulated with an average follicle size at the time of treatment of 36.85 ± 0.78 mm, significantly lower than non-treated jennies (39.89 ± 0.91 mm), whereas jennies that responded adequately to hCG had an average follicle size at the time of treatment not significantly different from any of the groups (37.85 ± 1.48 mm).

Our data show similar ovulation rates to previous studies using deslorelin acetate or hCG in mares [66], but lower than those reported in previous studies using 3000 IU of hCG [67] or 2.2 mg of deslorelin acetate [48] in jennies. The recommended dosage for hCG (in mares) is 1500–3000 IU. Although the complete vial of Veterin Chorion contains 3000 IU of hCG, only 1500 IU was used for ovulation induction in this study. This dosage was previously described for jennies by Serres et al. [68], and it was successfully used for inducing ovulation in jennies in later studies [61,69,70,71,72]. However, this is the first study in which this concentration of hCG is compared to 0.75 mg of deslorelin acetate for induction of ovulation in this species. It is possible that the doses of 0.75 mg of deslorelin acetate were too low to maintain a consistent LH release and, consequently, to induce timely ovulation in jennies. However, it has been previously demonstrated that a lower dose of deslorelin acetate (i.e., 0.1–0.2 mg) might be enough to induce timely ovulation [73]. In mares, it has been reported that 0.5 mg of deslorelin acetate is as effective as 1 mg of deslorelin acetate in inducing timely ovulation [43]. On the other hand, a recent study suggested that individual refractoriness to GnRH analogues exists [3]. Unfortunately, to our knowledge, no studies have been conducted yet about the efficacy of different doses of deslorelin acetate in jennies, and therefore further studies on this issue are needed in the future.

Regarding the low ovulatory response to hCG (60.9%; 14/23), this finding could be explained by differences in the size of the preovulatory follicle. Interestingly, the average size of the preovulatory follicle at the moment of hCG treatment was significantly higher in the jennies that responded to treatment (ovulation occurred within 48 h, 40.09 ± 1.48 mm) than in those that did not ovulate after 48 h of treatment (35.11 ± 1.86 mm). It is known that mares respond most consistently to hCG if the follicular size is ≥ 35 mm [38], a time at which the granulosa cell receptors respond to LH [74]. A reduced efficacy of hCG after repeated use in the same breeding season has also been described previously [31,39]. In our study, 6 of the 13 hCG-treated jennies received more than two consecutive treatment sessions, which might affect its efficacy. However, this is somewhat difficult to interpret in this study because only a limited number of jennies were treated more than twice. Altogether, both products used to induce ovulation resulted in acceptable response rates for routine use in donkey ET programs.

It has been suggested that in mares that ovulated within 24 h after treatment, either they responded exceptionally fast to the ovulation-inducing agent or, more probably, the dominant follicle may have already been under the influence of endogenous LH [41]. The rate of ovulation between 0 and 24 h was numerically higher but not significantly different in DES-treated (24.6%) compared to hCG-treated jennies (17.4%), which is consistent with that reported previously in mares [28]. Moreover, the percentage of hCG-treated jennies that ovulated within 24 h after treatment was similar to that described in other studies in mares (12.7–18.5%) [41,75].

It is noteworthy that the overall interval from hCG to ovulation (ITO) was longer than that observed in DES-treated jennies (62.61 ± 7.20 h vs. 48.79 ± 2.69 h). Moreover, we observed a peak in ovulations 24–48 h after DES treatment, similarly to previous studies [30,66,76]. Overall, our results suggest that both treatments have similar efficacy in inducing ovulation in treated jennies, but it seems that DES treatment could induce ovulation in a shorter and more predictable time window.

The overall incidence of double ovulations in induced estrus was surprisingly low (28.6%) compared to that reported in the literature [18,77], where estrus induction using PGF2α was associated with a higher occurrence of double ovulations or twin pregnancy. This difference could be explained by the influence of other factors such as age [18], breed [11] or body condition score [55]. The incidence of double ovulations in hCG-treated jennies was similar to that of DES-treated jennies (39.1% vs. 24.6%), which is in agreement with findings reported in mares [22]. Unfortunately, the increased ovulation rate for hCG-treated jennies did not result in a greater number of embryos recovered (hCG: 60.9% vs. DES: 72.1%).

The current study shows a lack of seasonal effect on ITO for both ovulation-inducing treatments (hCG and DES). This contrasts with reports in previous studies on mares, where ovulation occurred sooner after induction during the breeding season by administration of 1500–2000 IU of hCG [27,78] or 0.5 mg of buserelin acetate [79]. It is widely known that the reproductive cyclicity of jennies is less affected by season than that of horses and ponies [8,9,15,80], a fact which could explain our results. In contrast to previous studies in mares [79,81], the ovulation distribution was clearly different in DES-treated jennies, being lower in winter. This finding could be explained by the fact that nonresponder jennies took longer to ovulate (spontaneously), as reflected by the ITO.

Although no significant differences between ovulation-inducing agents were found for PFR, ERR and EG, the ERR obtained in DES-treated jennies was numerically higher than that of hCG-treated jennies (72.1% vs. 60.9%). This finding could be related to the ovulation rate observed in our study (DES: 78.7% vs. hCG: 60.9%). Moreover, ITO was significantly higher in hCG-treated than in DES-treated jennies (62.61 ± 7.20 h vs. 48.79 ± 2.69 h), and the phenomenon of aged oocytes from persistent follicles is widely recognized [82]. The present study, and previous studies on mares [83] have demonstrated that EG is similar between ovulation-inducing treatments. Together, our findings could suggest that the administration of DES to donor jennies may result in a greater number of recovered embryos.

The present study also demonstrates that follicle size at the time of treatment influenced the ITO, thereby supporting the hypothesis that smaller follicles (≤35 mm) require a longer time interval for ovulation induction, as they have fewer receptors for FSH and LH [74]. This finding is in consonance with that reported by Carluccio et al. [35,49] in Martina Franca donkeys, where a longer ITO was found when the follicular diameter at the time of treatment was ≤35 mm as compared to larger follicles (36–40 mm and >40 mm), and it was supported by the correlation analysis. On the other hand, we observed that PFR and ERR were numerically higher (*p* > 0.05) when ovulation was induced in larger follicles (>40 mm), whereas the opposite was found for EG. These findings could be due to the fact that the bigger the follicle, the closer the ovulation and, therefore, the higher the pregnancy rate [84]. However, further experiments are needed to confirm these results.

Abnormalities in the uterine edema pattern could help to predict the reproductive performance of treated donor jennies. Accordingly, EG significantly decreased with an increase in the uterine edema grade (≥3, range 0–4) at the time of treatment. Similarly, both PFR and ERR were numerically higher, but not significant, in jennies with a uterine edema score of 1. A low edema score probably indicates that luteinization is starting [50], shortening the time between breeding and fertilization, increasing the pregnancy rates. Although, to our knowledge, there are no previous studies evidencing the relationship between the EG and uterine edema patterns in jennies, a significant decrease in both embryo recovery and pregnancy rates was recorded in mares with excessive edema, pre- and post-mating [85,86]. In summary, our results could suggest that the presence of a uterine edema score of 3 at the time of treatment is not a good indication of induction of ovulation in jennies as it affects the EG.

## 5. Conclusions

Both prostaglandin treatments produced a satisfactory clinical response in jennies, but luprostiol led to a better synchronization of estrus and ovulation than dinoprost tromethamine, which was more marked in winter. However, the overall embryo recovery rate tended to be higher in dinoprost-treated than in luprostiol-treated jennies, without deleterious effects on embryo quality. The ovulation-inducing agents used in this study successfully hasten ovulation in jennies; however, deslorelin acetate seems to be more effective than hCG for inducing timely ovulation, particularly in summer and autumn. The overall embryo recovery rate was similar between treatments, but numerically higher in the deslorelin-treated jennies. Our study also demonstrates that the follicular diameter at treatment affected the interval from treatment to ovulation, being higher in small follicles (≤35 mm in diameter), but no effect on the embryo recovery rate was observed. Finally, the uterine edema pattern could be an optimal tool to predict embryo quality, since poor-quality embryos were collected in jennies that had high uterine edema scores.

## Figures and Tables

**Table 1:** Prostaglandin–ovulation interval (POI) and interovulatory interval (IOI) in cycles of jennies treated with luprostiol (LUP) or dinoprost tromethamine (DIN).

Group	Animals	Cycles	Age (Years)	POI (Days)	IOI (Days)
LUP	16	79	6.87 ± 0.37	9.73 ± 0.33	16.86 ± 0.34
DIN	14	33	6.76 ± 0.52	10.21 ± 0.57	17.73 ± 0.55
Total	19	112	6.84 ± 0.30	9.88 ± 0.29	17.12 ± 0.29

The lack of superscripts indicates no significant differences (NS; *p* > 0.05).

**Table 2:** Prostaglandin–ovulation interval (POI) and interovulatory interval (IOI) in cycles of jennies treated with luprostiol (LUP) or dinoprost tromethamine (DIN), depending on the season.

Season	Animals	Cycles	POI (Days)		IOI (Days)	
			LUP	DIN	LUP	DIN
Winter	10	13	8.88 ± 0.58 ^b^	11.40 ± 0.75 ^a^	16.25 ± 0.80	18.40 ± 0.51
Spring	13	52	9.94 ± 0.45	6.50 ± 0.50	17.16 ± 0.46	15.00 ± 1.00
Summer	7	8	9.00 ± 1.78	8.50 ± 1.56	16.75 ± 1.38	15.75 ± 1.49
Autumn	17	39	9.71 ± 0.62	10.59 ± 0.74	16.29 ± 0.64	18.18 ± 0.74
Total	19	112	9.73 ± 0.33	10.21 ± 0.57	16.86 ± 0.34	17.73 ± 0.55

^a,b^ Different superscripts indicate significant differences between prostaglandin treatments (LUP vs. DIN) within the interval (POI or IOI) (*p* < 0.05).

**Table 3:** Distribution of estrous cycles in jennies treated with luprostiol (LUP) or dinoprost tromethamine (DIN), depending on the prostaglandin–ovulation interval (POI; <9 days, 9–11 days, >11 days).

POI	Total Cycles	Group	
	*n* (%)	LUP	DIN
<9 days	41 (36.6%)	29 (36.7%) ^ab^	12 (36.4%)
9–11 days	42 (37.5%)	32 (40.5%) ^a^	10 (30.3%)
>11 days	29 (25.9%)	18 (22.8%) ^b^	11 (33.3%)
Total	112 (100%)	79 (100%)	33 (100%)

^a,b^ Different superscripts indicate significant differences between prostaglandin treatments (*p* < 0.05).

**Table 4:** Embryo recovery rate (ERR), positive flushing rate (PFR) and embryo morphological grade (EG, 1–4) for the luprostiol (LUP) and dinoprost tromethamine (DIN) groups.

Group	Positive Flushing Rate	Embryo Recovery Rate	Embryo Grade
LUP	48/79 (60.8%)	51/79 (64.6%)	1.27 ± 0.08
DIN	24/33 (72.7%)	27/33 (81.8%)	1.19 ± 0.09
Total	72/112 (64.3 %)	78/112 (69.6 %)	1.24 ± 0.06

The lack of superscripts indicates no significant differences between treatments (LUP vs. DIN) (NS; *p* > 0.05).

**Table 5:** Jenny age, follicular diameter and uterine edema score at time of treatment (0–4), interval to ovulation (ITO), percentage of jennies ovulating within 48 h and percentage of double ovulations after the administration of human chorionic gonadotropin (hCG) and deslorelin acetate (DES).

Group	Animals	Cycles	Age (Years)	Follicular Diameter (mm)	Uterine Edema Score	ITO (h)	% Ovulation within 48 h	% Doble Ovulations
hCG	13	23	6.83 ± 0.47	37.85 ± 1.27	2.56 ± 0.17 ^a^	62.61 ± 7.20 ^a^	60.9 (14/23)	39.1 (9/23)
DES	12	61	6.56 ± 0.45	36.34 ± 0.71	2.00 ± 0.12 ^b^	48.79 ± 2.69 ^b^	78.7 (48/61)	24.6 (15/61)
Total	19	84	6.63 ± 0.35	36.72 ± 0.62	2.13 ± 0.10	52.57 ± 2.83	73.8 (62/84)	28.6 (24/84)

^a,b^ Different superscripts indicate significant differences in each parameter between ovulation-inducing agents (*p* < 0.05).

**Table 6:** Distribution of ovulations (0–24 h, 24–48 h, >48 h) after the administration of human chorionic gonadotropin (hCG) and deslorelin acetate (DES) in jennies.

Group	Animals	Cycles	Ovulation Rates (*n*, %)		
			0–24 h	24–48 h	>48 h
hCG	13	23	4 (17.4%)	10 (43.5%)	9 (39.1%)
DES	12	61	15 (24.6%) ^b^	33 (54.1%) ^a^	13 (21.3%) ^b^
Total	19	84	19 (22.6 %)	43 (51.2%)	22 (26.2%)

^a,b^ Different superscripts indicate significant differences in the distribution of ovulations (*p* < 0.05).

**Table 7:** Effect of season on the interval to ovulation (ITO) and ovulation rate (OR) in cycles of jennies treated with human chorionic gonadotropin (hCG) and deslorelin acetate (DES).

Season	Animals	Cycles	ITO (h)		OR (%)	
			hCG	DES	hCG	DES
Winter	10	12	61.33 ± 14.84	64.00 ± 8.00	66.7	33.3 ^a^
Spring	13	34	56.00 ± 16.00	47.23 ± 3.24	33.3	74.2 ^ab^
Summer	7	9	*	45.33 ± 4.81	*	88.9 ^b^
Autumn	17	29	65.45 ± 9.21	45.18 ± 4.06	63.6	88.9 ^b^
Total	19	84	62.61 ± 7.20	48.30 ± 1.98	60.9	78.7

^a,b^ Different superscripts indicate significant differences (*p* < 0.05). * Missing data.

**Table 8:** Embryo recovery rate (ERR), positive flushing rate (PFR) and embryo morphological grade (EG: 1–4) in donor jennies treated with human chorionic gonadotropin (hCG) and deslorelin acetate (DES).

Group	Positive Flushing Rate	Embryo Recovery Rate	Embryo Grade
hCG	12/23 (52.2%)	14/23 (60.9%)	1.29 ± 0.16
DES	43/61 (70.5%)	44/61 (72.1%)	1.36 ± 0.09
Total	55/84 (65.5 %)	58/84 (69.0 %)	1.34 ± 0.86

The lack of superscripts indicates no significant differences between ovulation-inducing agents (NS; *p* > 0.05).

**Table 9:** Effect of follicular diameter at time of treatment (≤35 mm, 36–40 mm, >40 mm) on interval to ovulation (ITO), positive flushing rate (PFR), embryo recovery rate (ERR) and embryo morphological grade (EG) in jennies.

Follicular Diameter (mm)	ITO (h)	PFR	ERR	EG
≤35	64.30 ± 5.54 ^a^	21/32 (65.6%)	21/32 (65.6%)	1.38 ± 0.13
36–40	46.34 ± 3.75 ^b^	18/29 (62.1%)	18/29 (62.1%)	1.44 ± 0.15
>40	44.21 ± 4.21 ^b^	15/19 (78.9%)	15/19 (78.9%)	1.27 ± 0.15
Total	53.10 ± 2.95	54/80 (67.5%)	54/80 (67.5%)	1.37 ± 0.08

^a,b^ Different superscripts indicate significant differences (*p* < 0.05).

**Table 10:** Effect of uterine edema (grade: 0–4) at time of treatment on interval to ovulation (ITO), embryo recovery rate (ERR), positive flushing rate (PFR) and embryo grade (EG) in jennies.

Uterine Edema	ITO (h)	PFR	ERR	EG
0	48.00 ± 9.80	3/4 (75.0%)	3/4 (75.0%)	1.00 ± 0.00 ^a^
1	42.86 ± 4.49	10/14 (71.4%)	10/14 (71.4%)	1.20 ± 0.13 ^ab^
2	49.71 ± 4.77	18/28 (64.3%)	18/28 (64.3%)	1.17 ± 0.09 ^ab^
3	60.80 ± 5.72	21/30 (71.0%)	21/30 (71.0%)	1.71 ± 0.16 ^b^
4	72.00 ± 0.00	0/1 (0%)	0/1 (0%)	-
Total	52.99 ± 3.06	52/77 (67.1%)	52/77 (67.1%)	1.38 ± 0.08

^a,b^ Different superscripts indicate significant differences (*p* < 0.05).

## Data Availability

The data presented in this study are available upon reasonable request from the corresponding author.
